# The WHO Institutional Repository for Information Sharing–African Programme for Onchocerciasis Control Collection: an electronic resource of the Onchocerciasis Control Programme and African Programme for Onchocerciasis Control experience and data to inform onchocerciasis elimination strategies

**DOI:** 10.1093/inthealth/ihaf090

**Published:** 2026-06-01

**Authors:** Dziedzom K de Souza, Olabanji Surakat, Arwa Elaagip, Franklin Ayisi, Lassane Koala, Kareen Atekem, Charles D Mackenzie, Annette C Kuesel, Daniel A Boakye

**Affiliations:** Department of Parasitology, Noguchi Memorial Institute for Medical Research, College of Health Sciences, University of Ghana, Legon-Accra, Ghana; Department of Zoology, Osun State University, Osogbo, Nigeria; Department of Parasitology and Medical Entomology, Faculty of Medical Laboratory Sciences, University of Khartoum, Khartoum, Sudan; African Regional Postgraduate Programme in Insect Science (ARPPIS), University of Ghana, PMB LG 59, Legon, Accra, Ghana; Centre National de Recherche Scientifique et Technologique (CNRST)/ Institut de Recherche en Sciences de la Santé (IRSS), Département Biomédical et Santé Publique, Direction Régionale de l'Ouest, Bobo-Dioulasso, Burkina Faso; Department of Entomology, Center for Infectious Disease Dynamics, Pennsylvania State University, PA, USA; The Ending Neglected Diseases (END) Fund, New York, USA; UNICEF/UNDP/World Bank/WHO Special Programme for Research and Training in Tropical Diseases (WHO/TDR), Geneva, Switzerland; Department of Parasitology, Noguchi Memorial Institute for Medical Research, College of Health Sciences, University of Ghana, Legon-Accra, Ghana; The Ending Neglected Diseases (END) Fund, New York, USA

**Keywords:** APOC, APOC Collection, OCP, onchocerciasis, WHO IRIS

## Abstract

**Background:**

As countries progress from control of onchocerciasis as a public health problem to elimination of parasite transmission, more evidence on vector-related activities and transmission assessments is needed to inform WHO guidelines and country strategies to achieve the targets of the 2030 Neglected Tropical Disease Roadmap. For vector-borne diseases, entomology plays a critical role in the elimination and verification of transmission based on vector infectivity. The vector control-based Onchocerciasis Control Programme (OCP) in West Africa, 1974–2002, had accumulated relevant experience and data. The African Programme for Onchocerciasis Control (APOC; 1995–2015) extended onchocerciasis control, based primarily on mass ivermectin administration, to all African endemic countries and supported many implementation research projects. OCP and APOC documents, previously available only in hard copy from OCP or APOC, are now accessible in the WHO Institutional Repository for Information Sharing (WHO IRIS). Most documents cover numerous topics and include large amounts of unpublished data. Here, we provide an overview of the OCP and APOC documents in IRIS.

**Methods:**

We reviewed and summarised the documents in WHO IRIS as a resource to guide current elimination efforts. We also provided examples for potential uses of the information for mapping, diagnostics, entomology and social science research to the preparation of elimination dossiers.

**Results:**

The APOC Collection included 8150 documents. Of these, 3895 were in English and 4194 in French. Topics covered in the documents include community-directed treatment with ivermectin, cytotaxonomy, diagnosis, drug effectiveness, environmental modification, epidemiology, health economics, impact assessment, insecticide resistance, larviciding, mapping, programme implementation, slash and burn, transmission assessment and vector control.

**Conclusion:**

The APOC Collection in WHO IRIS makes the wealth of OCP and APOC experience and data available to the onchocerciasis control/elimination and research community.

## Introduction

Community-directed treatment with ivermectin (CDTi) is currently the principal intervention for onchocerciasis. Contrary to initial expectations, studies in Senegal and Mali suggested the feasibility of eliminating onchocerciasis using CDTi in Africa.^1,2^ Subsequent evaluations of infection prevalence in areas under long-term CDTi in countries within the mandate of the African Programme for Onchocerciasis Control (APOC, 1995–2015)^3,4^ showed prevalences consistent with elimination having been achieved or being achievable. This led to a paradigm shift in the WHO and African countries' objectives, from the elimination of onchocerciasis as a public health problem to the elimination of transmission of the parasite *Onchocerca volvulus*. This change was discussed in 2010 at the 16th session of the Joint Action Forum of APOC^5^ and is now reflected in the WHO/Neglected Tropical Disease 2030 Roadmap.^6^

Entomology plays a crucial role in the elimination of vector-borne diseases. Understanding how the vectors influence transmission is key to elimination activities. Identifying the vector species and distribution in disease-endemic areas helps to effectively target elimination efforts.^7,8^ The *Simulium* vectors of *O. volvulus* encompass different species in the *Simulium neavei* group and the *Simulium damnosum* complex that play a role in transmission in different areas. Regular surveillance helps monitor blackfly populations, their distribution, as well as infection and infectivity rates.^9–11^ These data are essential for assessing the risk of transmission and the impact of ongoing interventions. In areas under CDTi, this information helps to optimise the distribution of ivermectin. The WHO entomology manual emphasises vector infectivity as a measure for the level of residual transmission that supports stopping CDTi.^12^ Based on entomological findings, targeted vector control strategies can be implemented as required. These may include the larviciding of breeding sites and environmental management (‘slash and burn’) to reduce fly populations. Entomology provides essential scientific insights to guide and improve onchocerciasis elimination programmes, aiming for a world free of this debilitating disease.

The strategies of the APOC (and of the Onchocerciasis Elimination Program for the Americas, 1991 to date) were mainly or exclusively based on mass drug administration of ivermectin, implemented in the APOC countries via CDTi. While control of onchocerciasis as a public health problem was the objective, entomological expertise, understanding of vector biology and information on the vector in the endemic areas was minimal, leading to a decline in entomological knowledge and data collection.^13,14^ By contrast, the strategy of the Onchocerciasis Control Programme (OCP) in West Africa (1974–2002) was based on vector control. The large amounts of entomological knowledge and data accumulated by the OCP will be valuable in forming elimination efforts across Africa.

The OCP undertook research to guide its vector control activities. Further, it supported research in external institutions to resolve challenges to control activities such as insecticide resistance, vector migration and species identification. The OCP worked with experts on an ad-hoc basis and as members of its external technical advisory committee, the ‘Expert Advisory Committee’ (EAC), which advised on the basic and operational research conducted and/or funded by the OCP. The EAC reviewed the results of the study and regularly monitored the effect of vector control operations as a basis for advising on OCP operations and new research needs. Most of the work was not published but was summarised in OCP reports, thus was not generally accessible. Starting during the preparation of the closure of APOC, the WHO digitised all OCP and APOC documents. They are now publicly available in the WHO Institutional Repository for Information Sharing (WHO IRIS), in what is referred to as the APOC Collection (https://apps.who.int/iris/handle/10665/274421). Documents are primarily available in English or French and some in both languages.

The wealth of OCP and APOC experience and data in the APOC Collection can support the decision-making processes of Ministries of Health, the guidance provided by National Onchocerciasis Elimination Committees and assist in the preparation of dossiers for WHO verification of interruption of transmission. We reviewed the APOC Collection to combine the documented experience and data within peer-reviewed publications to address challenges to sustainable elimination of *O. volvulus* transmission and its verification within countries and across borders. This paper provides an overview of the topics covered in the APOC collection documents and describes potential uses to support evidence-based planning for onchocerciasis elimination programmes.

## WHO IRIS

The WHO IRIS (https://iris.who.int/) is an electronic repository of documents published by the WHO. It is continuously updated as new publications become available. For each record an identification number, authors, place of publication, year of publication, hyperlink to the document, number of pages, language, publisher, Medical Subject Headings (MeSH) headings, title, type of publication (e.g. technical document, governing body document, meeting report) and identification number of the document in other languages (if applicable) is available. For some records, MeSH qualifiers and subjects or keywords are included. WHO IRIS has full-text search capacity (videos and an advanced search user's guide are available under ‘Help’). This allows, for example, searching by country name. Searching can be done across all of IRIS or within selected ‘communities’ (e.g. Headquarters, Regional Office for Africa; https://iris.who.int/community-list) and ‘collections’ (e.g. ‘African Programme for Onchocerciasis Control [APOC]’, ‘AFRO Country Cooperation Strategies’ within the community Regional Office for Africa). The results of searches can be exported as CSV, Excel, BibTex or RIS files. The maximum number of records per export is 500. The citation is provided for each document, which can be exported as a BibTex or RIS file for import into reference manager software.

## APOC Collection search and results

**Figure 1.**
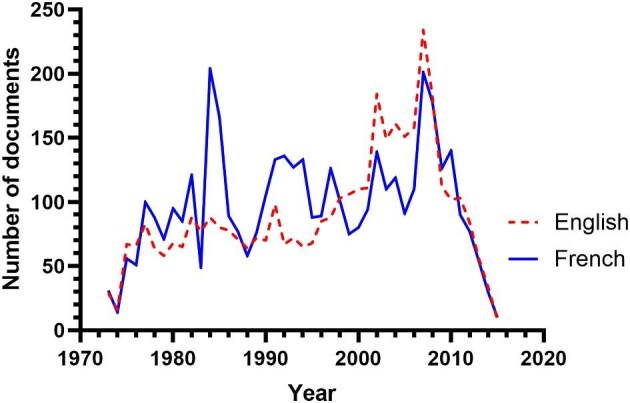
Number of records in the APOC Collection by year of publication. APOC: African Programme for Onchocerciasis Control.

As of the last search in July 2024, the APOC Collection (https://iris.who.int/handle/10665/274421) in WHO IRIS included 8150 documents. Of these, 3895 were in English and 4194 in French, including ‘duplicates’ (i.e. available in both languages). Figure 1 shows the number of records by year of publication. For comparison, a PubMed search on 19 December 2024 for articles with onchocerciasis in the title or abstract published from 1974 to 2015 and from 1911 to 2024 gave 2749 and 4298 records, respectively.

**Figure 2.**
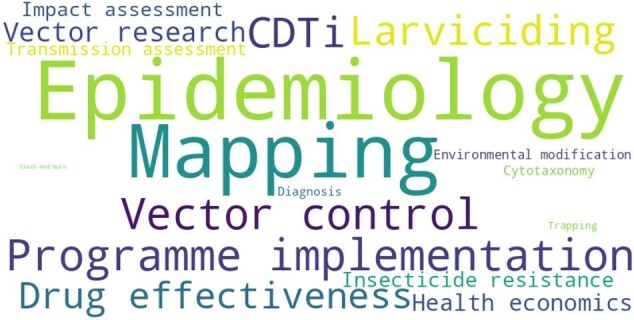
Word cloud analysis of the results of the full-text search of the APOC Collection. APOC: African Programme for Onchocerciasis Control.

To appreciate the information in these documents, a list of keywords intended to cover significant aspects of onchocerciasis control and elimination efforts (Table 1) was developed and used for full-text search of the APOC Collection. Using these keywords, the IRIS full-text search capacity was used to identify the number of documents available with the relevant information (Table 1). Figure 2 shows the word cloud analysis of the search results. It should be noted that many documents include content fitting several keywords and may also cover topics not included in Table 1. For example, the ‘Report of the thirty-fifth session of the Technical Consultative Committee’^15^ discusses topics linked to Impact Assessment, CDTi, Training (development and implementation of a community-directed intervention curriculum), Programme implementation, Transmission assessment, Diagnostics/test performance, Epidemiology, Trapping, Mapping, Vector research, Cytotaxonomy, Feasibility of Onchocerciasis elimination, Cross border issues, Oncho-LF programme collaboration/co-implementation, ivermectin Response Markers/ivermectin resistance and moxidectin.

**Table 1.** Keyword search results. Note that the sum is larger than the number of documents because documents can cover multiple topics (including those not listed here)

Keyword	Number of documents in the APOC Collection
CDTi	2222
Cytotaxonomy	421
Diagnosis	363
Drug effectiveness	1903
Environmental modification	396
Epidemiology	3151
Health economics	1404
Impact assessment	1042
Insecticide resistance	1085
Larviciding	2165
Mapping	2537
Programme implementation	2449
Slash and burn	6
Transmission assessment	964
Trapping	277
Vector control	2391
Vector research	1670

APOC: African Programme for Onchocerciasis Control; CDTi: community-directed treatment with ivermectin.

## Potential use cases of the APOC Collection

Table 1 shows the diversity of information available in the collection that national onchocerciasis elimination programmes and researchers can exploit. Below are some examples of how the APOC Collection can be used.

### Mapping

**Figure 3.**
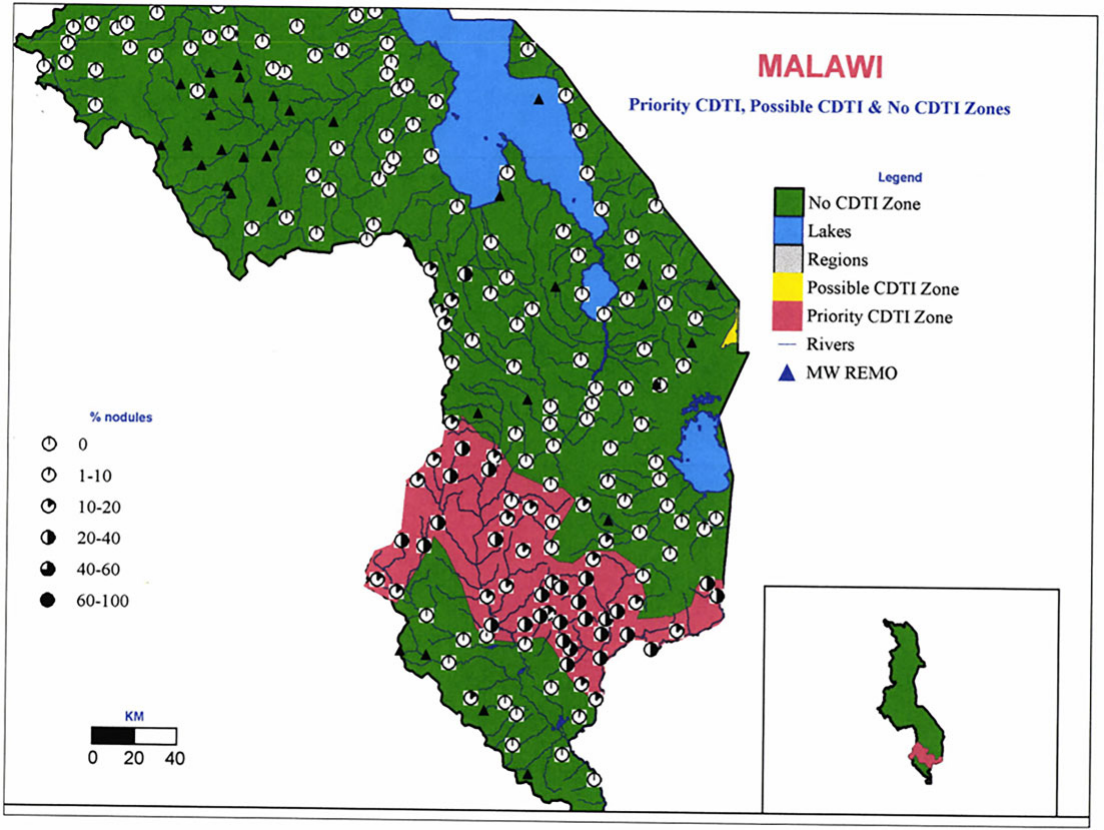
REMO map for southern Malawi, extracted from the WHO/APOC report.^23^ APOC: African Programme for Onchocerciasis Control; CDTi: community-directed treatment with ivermectin; MW: malawi; REMO: rapid epidemiological mapping of onchocerciasis.

**Figure 4.**
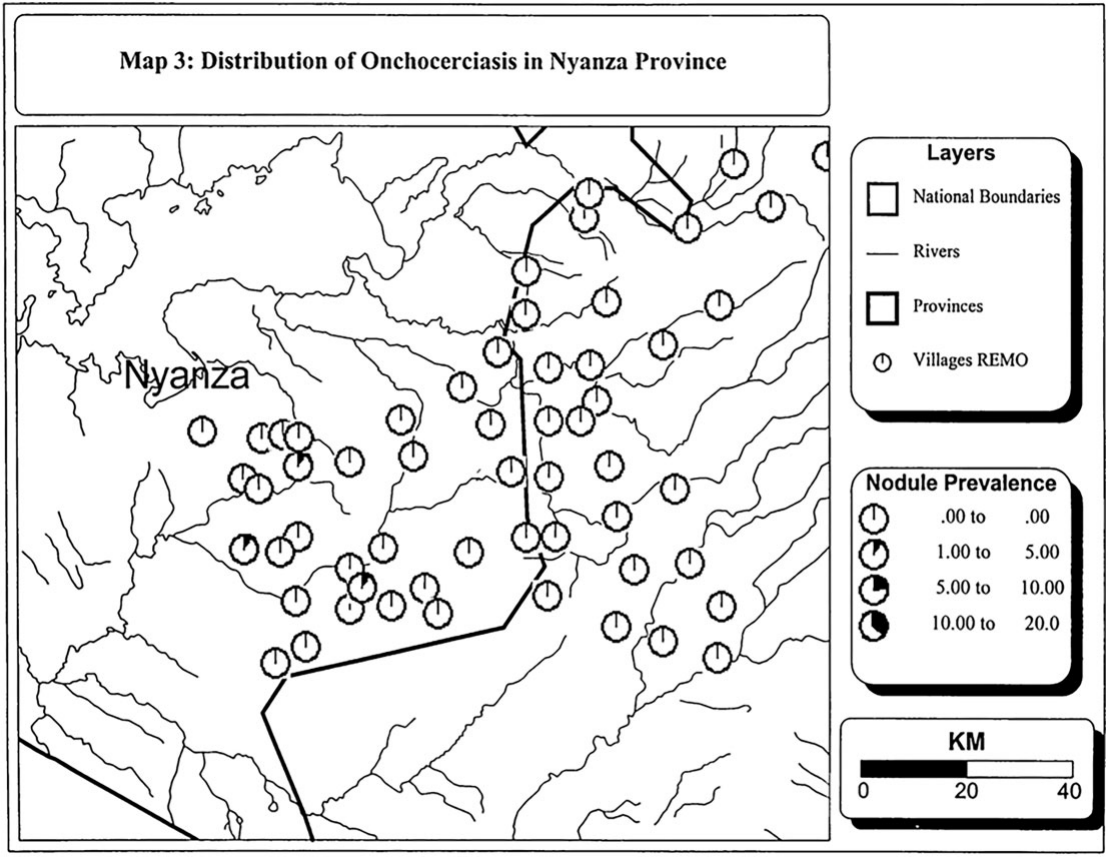
REMO map for Nyanza Province, Kenya, extracted from the WHO/APOC report.^24^ APOC: African Programme for Onchocerciasis Control; REMO: rapid epidemiological mapping of onchocerciasis.

The evolution from the control of onchocerciasis in Africa as a public health problem (i.e. interventions targeting only meso- and hyperendemic areas) to the elimination of parasite transmission (needing consideration of all endemic areas) requires a better understanding of the distribution of infection, in particular hypo-endemic areas.^16^ As a result, onchocerciasis elimination mapping (OEM) is currently recommended by the WHO Onchocerciasis Technical Advisory Subgroup to determine where treatment is required in potentially endemic areas not under treatment.^17^ The mapping information in documents in the APOC Collection could serve as a starting point for OEM. APOC conducted rapid epidemiological assessment (REA; based on prevalence of palpable onchocercal nodules) and rapid epidemiological mapping of onchocerciasis (REMO) prevalence^18,19^ in all countries within its mandate to identify hyper- and meso-endemic areas for CDTi implementation.^18–20^ It is essential to realise that the geographical unit for designating so-called ‘CDTi-priority areas’ was the country health system administrative borders to facilitate CDTi implementation, not individual communities. Information on nodule prevalence in separate communities in areas not considered needing CDTi for control of onchocerciasis as a public health problem can help identify areas for OEM. Figure 3 demonstrates this based on the information in the reports of the REA/REMO results in Malawi^21–23^: it shows communities in the ‘No CDTi zones’ where nodule prevalence was 10–40%. Such communities could be targeted for OEM. Similar data exist for other countries, including Kenya (Figure 4), where it is believed that onchocerciasis has been eliminated.^24^ Figure 4 shows three communities with nodule prevalence of 1–5%. Given the categorisation of prevalences in REMO maps, the raw nodule prevalence obtained in each community included in REA should be consulted. In many cases, these data are available in the tables provided in the reports. However, their use should consider that the epidemiological situation may have changed substantially since REA was conducted due to climate and anthropogenic effects and the sensitivity and specificity of nodule palpation, particularly in areas with low prevalence.^25,26^ Further, reviewing the data alongside information available in the expanded special project for elimination of neglected tropical diseases (ESPEN) database would be necessary for comprehensive assessments.

### Preparation of dossiers in support of requests for WHO verification of elimination of *O. volvulus* transmission

The presence of areas where onchocerciasis transmission is ongoing and where CDTi has not been previously implemented poses significant challenges to onchocerciasis elimination efforts. When countries progress towards preparing a dossier supporting a request for the WHO to verify elimination, they will need to have data or a strong rationale to support why an area was declared non-endemic. According to the 2016 ‘WHO Guidelines for Stopping Mass Drug Administration and Verifying Elimination of Human Onchocerciasis’, dossiers should contain a justification, rationale and criteria used to determine the geographical extent of the areas affected by the disease and to confirm the absence of transmission in areas outside the known affected areas.^27^ Many reports from the OCP and APOC contain country-level information that can inform elimination dossiers, and country programmes should ideally assemble and consider this information even before the decision to stop CDTi.

### Vector identification and migration

In particular, the OCP reports in the APOC Collection provide information on the vectors of onchocerciasis. The series of OCP reports on the cytotaxonomy of *Simulium damnosum* complex^28,29^ from 1975 to 1997 present details on the different species over time, making it a valuable resource based on which current cytotaxonomic identification can be compared to assess the presence of new vectors or changes in vector composition. During the OCP, many studies were also conducted on the reinvasion of *S. damnosum* that can provide insight relevant for addressing current cross-border transmission challenges.^30–33^

### Operational research

Further use of the REA data may be compared with current OEM data from selected countries to assess the added value of OEM to elimination efforts. Such comparison, which needs to consider the relative sensitivity and specificity of the methods used,^26^ may validate the need or otherwise of OEM and provide information for cost-saving measures towards OEM activities.

## Conclusion

The APOC Collection in WHO IRIS makes the wealth of OCP and APOC experience and data available to the onchocerciasis control/elimination and research community. Much of this information is in country reports and technical documents. While the uploading of OCP and APOC documents appears to have been completed, WHO IRIS is constantly updated with new WHO documents as they become available. The effective utilisation of this resource can help us use the lessons from approximately 40 y of onchocerciasis control efforts in Africa to address the current diverse needs of the community, thereby supporting progress towards elimination of *O. volvulus* transmission. This paper provides some potential uses of the APOC Collection in supporting OEM, vector studies and other programmatic activities. Even considering that many documents are duplicates (i.e. available in English and French), the number of documents is so large that their effective exploitation requires significant time, more than peer-reviewed publications because an ‘abstract’ is not usually available. To facilitate exploitation, the UNICEF/UNDP/World Bank WHO Special Programme for Research and Training in Tropical Diseases (TDR) funded the review of the APOC Collection summarised here.

## Data Availability

All data is available online in the WHO IRIS repository (https://iris.who.int/).

## Copyright

World Health Organization 2025. Open Access. This article is licensed under the terms of the Creative Commons Attribution 3.0 IGO License, which permits use, sharing, adaptation, distribution and reproduction in any medium or format, as long as you give appropriate credit to the WHO, provide a link to the Creative Commons licence and indicate if changes were made. The use of the WHO's name, except in reference to the article, and the use of WHO's logo, is not authorised as part of this licence. The link provided below includes additional terms and conditions of the licence. The images or other third party material in this article are included in the article's Creative Commons licence, unless indicated otherwise in a credit line to the material. Suppose material is not included in the article's Creative Commons licence and your intended use is not permitted by statutory regulation or exceeds the permitted use. In that case, you must obtain permission directly from the copyright holder. To view a copy of this license, visit http://creative commons.org/licenses/by/3.0/igo/.
